# Using extreme gradient boosting for predictive urban expansion analysis in Rustenburg, South Africa from 2000 to 2030

**DOI:** 10.1038/s41598-025-04304-w

**Published:** 2025-05-30

**Authors:** Paidamwoyo Mhangara, Eskinder Gidey, Bruce Steadman Mayise

**Affiliations:** 1 School of Geography, Archaeology and Environmental Studies, Faculty of Science, University of the Witwatersrand, Private Bag: 2050, Johannesburg, Gauteng South Africa; 2 Department of Land Resources Management and Environmental Protection (LaRMEP), College of Dryland Agriculture and Natural Resources, Mekelle University, P.O Box: 231, Mekelle, Tigray Ethiopia

**Keywords:** Urban expansion, Google earth engine, Extreme gradient boosting model, Rustenburg, South Africa, Environmental impact, Sustainability

## Abstract

Since the end of apartheid in 1994, South Africa has experienced significant urban population growth, with major cities undergoing particularly rapid expansion. Understanding spatial urban expansion in developing cities, such as Rustenburg, is essential for sustainable infrastructure planning, environmental management, and service provision. This study modeled the spatial extent of urban growth in Rustenburg from 1994 to 2022 using Extreme Gradient Boosting (XGB) and predicted future urban expansion from 2022 to 2030 through the Cellular Automata Simulation in the MOLUSCE plugin. The results indicate that in 1994, water bodies covered 88,303 hectares, developed areas covered 125,763 hectares, and vegetation occupied 2,513,336 hectares. By 2022, vegetated regions covered 5,104,145 hectares, developed areas increased to 586,017 hectares, and mining zones expanded to 113,224 hectares. Furthermore, the landscape featured disjointed urban areas, varied natural ecosystems, and significant fragmentation in undeveloped zones. In 2022, the number of water patches increased to 27,845, built-up areas to 60,690, vegetated areas to 102,119, bare land to 80,921, and mining areas to 23,296 patches. The Kappa statistics ranged from 0.93 (93%) to 0.99 (99%), demonstrating high reliability in simulating urban growth patterns. An overall accuracy of 95% and a Kappa statistic of 0.93 are indeed higher than the mean scientifically accepted accuracy level of 85% for urban growth analysis. These findings highlight significant changes in land use and landscape fragmentation over time and provide critical insights into urban growth patterns and their implications for water security, sustainable development, and community livelihoods in Rustenburg, South Africa. By examining these patterns, we can better plan for sustainable infrastructure, manage environmental impacts, and improve service provision in rapidly expanding urban areas.

## Introduction

Urbanization has profoundly transformed land use and land cover (LULC). In developed nations, migration propels urban development, while in underdeveloped countries, rapid population growth and migration serve as the primary drivers^1,2^. Furthermore, the growing influx of migrants, enterprises, and real estate developments demands additional resources in urban areas. Urban growth refers to the physical expansion and demographic increase of urban regions, often converting rural or undeveloped land into developed areas characterized by infrastructure, buildings, and human habitation. However, this expansion, both horizontally and vertically, results in challenges such as food scarcity, informal settlements, pollution, environmental degradation, and unemployment. In addition, planners’ failure to anticipate such growth patterns frequently leads to significant expansion and urban sprawl oversight. Understanding the dynamics and trends that influence our experience of the urban environment is essential, as numerous spatial–temporal connections depend on these patterns. Given that urbanization constitutes a significant environmental challenge globally, it is imperative to identify and track evolving patterns of urban expansion. Moreover, remote sensing and GIS have proven useful and cost-effective for understanding how land use changes over time and space, especially compared to more traditional socioeconomic indicators like population growth^2,3^. These technologies offer a broader view, immediate data collection, and various spectral properties.

Recently, the advantages of acquiring multiple monitoring stations in a short update cycle have established remote sensing as the most cost-effective method for obtaining precise LULC categorization^4^. Previous research has primarily used satellite imagery, such as MODIS, for regional LULC classification. However, the coarse spatial resolution (250–1000 m) of MODIS images is insufficient for reliable LULC maps due to the impact of mixed pixels. Instead, satellite data with higher spatial resolution, such as LANDSAT (30 m) and SPOT (20 m), have provided reliable solutions for LULC identification studies^4^. For instance, according to Patel et al.^5^, Landsat imagery is effective for assessing global urbanization trends over time. One of the most important remote sensing instruments available today is Google Earth Engine (GEE). Several researchers, such as Gidey et al.^3^, Patel et al.^5^, and Mhangara et al.^6^ used data from the Landsat Program and NASA to construct GEE, an online environmental data monitoring platform. GEE analyzes more than 40 years of Landsat data using cloud computing technology. Consequently, urban growth studies benefit from the platform’s rapid global data analysis, making it a useful tool for mapping and monitoring LULC. Therefore, it is imperative to meticulously examine the underlying causes and possible ramifications of this trend regarding resource availability and environmental sustainability.

For example, South Africa’s population increased by 37.2% over 20 years, from 40.58 million in 1996 to 55.7 million in 2016^7^. Furthermore, post-1994 economic growth in South Africa has spurred technological and infrastructure development, potentially exacerbating local air pollution and rural–urban mobility issues. A range of factors contribute to migration, including greater job opportunities, better access to healthcare and education, and the chance of higher salaries^7^. However, this has created significant risks to the environment, sustainable development, and the ecosystem. Over the last few decades, Rustenburg’s urban expansion has been exponential. Due to its abundant mineral resources, the natural environment is under increasing strain from large-scale industrialization^8^. In particular, mining activity in open-pit sites has grown, and the rising number of job seekers has fueled urbanization. As a result, increased mining, agricultural, and urban development have severely harmed the terrain^8^.

Additionally, the integration of robust models such as XGB (Extreme Gradient Boosting) and MOLUSCE (Modules for Land Use Change Evaluation) in urban growth analysis and prediction studies is a novel approach. It leverages the predictive capabilities of XGB and the spatial–temporal modeling strengths of MOLUSCE, resulting in more accurate and detailed findings of urban expansion. According to Gidey et al.^1^, utilizing these advanced tools together, researchers can better understand and anticipate urbanization patterns, providing valuable insights for sustainable urban planning and policymaking. This study emphasizes the importance of the XGB model and GEE in analyzing urbanization trends in rapidly developing cities like Rustenburg, providing valuable insights into the city’s urban growth over the past three decades. Therefore, it is critical to model, analyze, and track urban development in the study area. The aim of this study is to: (i) analyze the spatial extents of urban growth in the City of Rustenburg between 1994 and 2022 using XGB, a robust machine learning model; (ii) predict the future expansion of urban growth from 2022 to 2030 using the MOLUSCE (Modules of Land Use Change Evaluation) plugin’s Cellular Automata Simulation. The findings of this study are crucial for urban planning, policymaking, and sustainable development. This will help policymakers and environmentalists understand all the elements that contribute to urbanization. Moreover, improved mapping and monitoring of urbanization will enhance sustainable and environmental policies, mitigating the impacts and environmental degradation associated with urban expansion.

## Method

### Study area

**Fig. 1:**
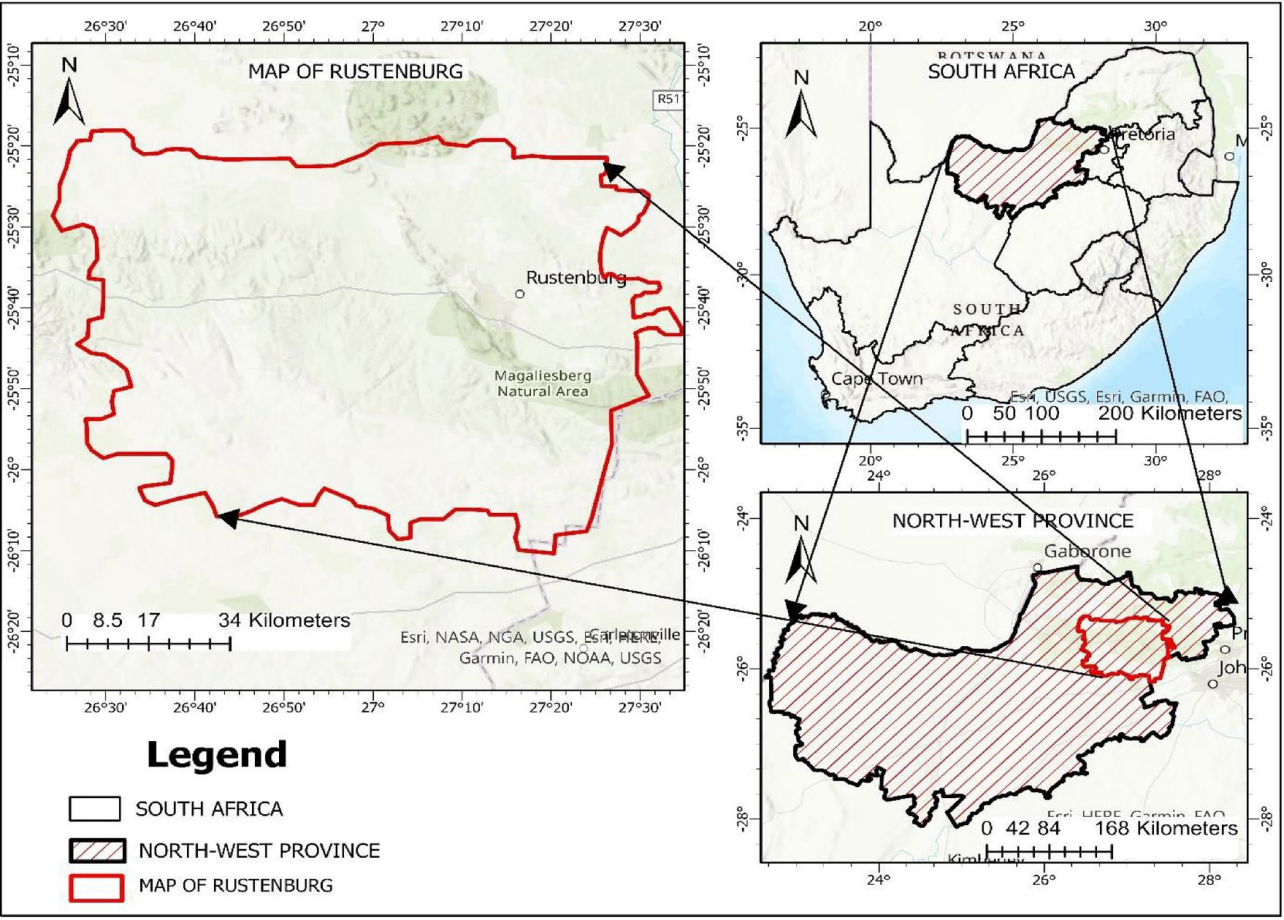
Study area location mapped with ESRI ArcGIS Pro 3.3.2 (Licensed from ESRI).

The study area is situated near the base of the Magaliesberg mountain range in South Africa’s Northwest Province (Fig. 1). Geographically, it is located at 25°40′0" south and 27°15′0" east, with a maximum elevation of 1170 m (Mudau et al., 2014). Rustenburg, a local town with a population of 549,600, is home to three of the world’s largest platinum mines, attracting migrants seeking employment in the mining industry. The mining sector accounts for an impressive 77% of the city’s GDP^9^. Rustenburg is in South Africa’s Highveld climate zone, characterized by hot, humid summers, pleasant days, and cold winter nights^7^. The city’s soils are loamy, rich in deep clay, and have modest infiltration and water-holding capacity. Rustenburg, which covers 3430 square kilometers, has expanded its ward count from 34 to 38 in response to strong population growth, new residential developments, and high population densities. The city’s economy is diverse, encompassing sectors such as agriculture, industry, retail, and tourism. Significant urbanization has occurred in recent years, with the construction of sophisticated shopping malls, residential developments, and corporate parks. The city’s economic success is intrinsically linked to the platinum mining industry, which accounts for 77% of its Gross Geographic Product (GGP). Rustenburg mines generate more than 70% of the world’s platinum^9^. In 2013, Rustenburg’s population was 242,892. The city attracts people from across South Africa and the Southern African region in search of job opportunities offered by mining operations. Its proximity to Gauteng, South Africa’s most populous province, is a major factor driving its rapid population growth, resulting in the establishment of both formal and informal communities within and around the city. Therefore, the rapid urbanization and extensive mining activities in Rustenburg underscore the importance of analyzing urban growth patterns to ensure sustainable development and effective management of natural resources such as land, water, and minerals.

### Data acquisition

This study utilized Landsat 5 TM with seven bands, Landsat 7 ETM, and Landsat 8 OLI with eleven bands for the periods 1994, 2000, 2006, 2012, 2018, and 2022 (Table 1). We collected the images annually, specifically between October 1st and November 1st. The seasonal variations in Rustenburg, which span from October to November and continue through March and April, determined the selected time frame. Conversely, image quality issues were the primary factor in selecting Landsat satellite image dates, prioritizing those with minimal or negligible cloud cover. We classified all the data sets as Collection 2 Tier 1 to identify and monitor changes in LULC. GEE offers atmospherically adjusted images, ready for classification. GEE provides users with quick and easily accessible online access to a vast collection of stored Landsat data^10^. Landsat images are freely available, cost-effective, and provide excellent resolutions in terms of space, time, and spectral characteristics, making them valuable for analysing time series data.

**Table 1:** Landsat image characteristics for the study.

Sensor	Wavelength (μm)	Spatial resolution (meters)
Landsat 4–5 Thematic Mapper (TM) (1994–2012)		
Band 1—Blue	0.45–0.52	30
Band 2—Green	0.52–0.60	30
Band 3—Red	0.63–0.69	30
Band 4—Near Infrared (NIR)	0.76–0.90	30
Band 5—Shortwave Infrared (SWIR) 1	1.55–1.75	30
Band 6—Thermal	10.40–12.50	120 (resampled to 30)
Band 7—Shortwave Infrared (SWIR) 2	2.08–2.35	30
Band 8—Panchromatic	0.52-.90	15
Landsat 8 Operational Land Imager (OLI) and Thermal Infrared Sensor (TIRS) (2018–2022)		
Band 1—Coastal aerosol	0.43–0.45	30
Band 2—Blue	0.45–0.51	30
Band 3—Green	0.53–0.59	30
Band 4—Red	0.64–0.67	30
Band 5—Near Infrared (NIR)	0.85–0.88	30
Band 6—Shortwave Infrared (SWIR) 1	1.57–1.65	30
Band 7—Shortwave Infrared (SWIR) 2	2.11–2.29	30
Band 8—Panchromatic	0.50–0.68	15
Band 9—Cirrus	1.36–1.38	30
Band 10—Thermal Infrared (TIRS) 1	10.6–11.19	100 (resampled to 30)
Band 11—Thermal Infrared (TIRS) 2	11.50–12.51	100 (resampled to 30)

### Preprocessing

#### Application of GEE and XGBoost Models for Satellite Image Processing, Classification, and Analysis

The largest challenges in remote sensing, particularly when utilizing Landsat imagery, are errors in multi-temporal satellite images generated by the recording sensor or missing data due to clouds and shadows^11^. Therefore, performing image preprocessing is important. It is necessary to conduct atmospheric, radiometric, and geometric evaluations during the preprocessing stage to predict the reflectance to the ground, which necessitates applying atmospheric correction. To mitigate the effects of clouds and their shadows, we use the GEE cloud score method. This method assigns a score to each pixel based on its likelihood of being clear or cloudy, helping to improve the accuracy of our analyses. GEE, a sophisticated tool, enables global visualization and analysis of geospatial data. By processing and analyzing satellite imagery, this platform provides users with access to a vast library of satellite images, including those from Landsat 4–5 TM, Landsat 7 ETM, and Landsat 8 OLI. Analysts frequently use these databases to monitor urban growth and analyze changes in land cover and land use. They are extremely useful tools for evaluating accuracy due to their spatial resolution quality and temporal coverage. This method produces a basic cloud likelihood score, ranging from 0 to 100, by combining brightness, temperature, and the Normalized Difference Snow Index (NDSI)^10^. We used the filtered collection by date feature of the GEE archive to select data for analysis. After filtering by year, we assembled the image. The final output was based on each pixel’s median value for each year. We randomly selected 400 samples for each class. Table 2 provides a comprehensive description of various land cover types. For the LULC classification, the sample points in the study region were divided into two groups: training samples (70%) and testing samples (30%). We constructed the predictive model using training samples and verified its performance using testing samples.

**Table 2:** Illustrates the various types of land cover that are described in the study area.

Land cover type	Description
Bare land	Barren landscapes, such as deserts, rocky terrain, or recently disturbed land, are characterized by a lack of substantial vegetation cover
Vegetated	These are areas covered by various types of vegetation, including forests, grasslands, and shrublands
Urban	Urban land cover consists of developed areas such as towns, cities, and roads, as well as infrastructure
Water bodies	This classification comprises both naturally occurring water features, such as reservoirs, lakes, and oceans, and man-made structures, including rivers
Mining area	These are areas characterized by the presence of mining operations, including quarries, open-pit mines, and other extraction sites

XGBoost uses an extreme gradient boosting framework that leverages the benefits of parallel tree boosting. To address the issue of overfitting, it utilizes a regularization technique that is more advanced than traditional gradient boosting, leading to enhanced performance^12,13^. The current parameter settings for XGBoost were chosen to optimize performance and efficiency. These settings were derived from a combination of previous studies, experimental optimization, and specific criteria tailored to the dataset and problem at hand. The boosting strategy combines weak learners to form a strong learner, enhancing predictive power^12^. Parallel processing speeds up training, making it suitable for large datasets^14^. The histogram-based approach for finding optimal splits reduces computational complexity, improving performance. Weak learners have low correlation with the target variable, while strong learners have high correlation. Combining weak learners creates a robust model^15^.

XGBoost combines many weak learners to predict outcomes using a boosting strategy^12^. This methodology efficiently expedites procedures and is suitable for both regression and classification tasks^14^. The terms “weak” and “strong” refer to the extent to which classification learners’ correlations with the actual target variable are lacking or robust^15^. XGBoost employs a histogram-based approach that is pre-sorted and utilizes parallel processing to identify optimal splits. The methodology uses a leaf-wise pruning technique, which involves examining each decision tree leaf to find the best solution, and the gradient descent procedure to minimize errors^16^.

1 $$OF\left(\theta \right)=\sum_{i=1}^{n}l\left({y}_{i},{\overline{y} }_{a}\right)a+\sum_{k=1}^{K}\Omega ({f}_{k})$$

where $\sum_{i=1}^{n}l\left({y}_{i},{\overline{y} }_{a}\right)$ represents the model fits to the training data using the loss function of the root mean square error. $a$ represents the previous ensemble prediction contribution to current iteration prediction (θ). $\sum_{k=1}^{K}\Omega ({f}_{k})$ represents the regularization term that prevents overfitting. $K$ indicates the number of individual trees. ${f}_{k}$ is one of the ensemble’s trees (or tree in the ensemble). ${y}_{i},{\overline{y} }_{a}$ are the actual and expected results of the class.

The robust approaches we have applied help us analyze the spatial extents of urban growth in the City of Rustenburg between 1994 and 2022 and predict future expansion from 2022 to 2030. These methods enhance our analysis of urban dynamics and LULC changes, directly addressing our specific research objectives. By employing these techniques, we can effectively assess changes in land use and land cover, providing valuable insights into urban growth patterns.

#### Model verification and accuracy assessment

In this study, we used a confusion matrix to verify and assess the accuracy of our models and findings. The confusion matrix is a tool that helps us evaluate the performance of our classification models by comparing the predicted values with the actual values, allowing us to identify any discrepancies and measure the overall accuracy. The output results of machine learning models are less significant without validation; therefore, accuracy is a crucial step^17^. When validating map classification, the confusion matrix (CM) is a critical tool^18^. The confusion matrix contrasts the ground reference with the predicted class label. The confusion matrix allows for the determination of accuracy metrics such as overall accuracy, Kappa, and user precision^18^. As a result, it is an indispensable tool for land use and land cover research. Accuracy assessment provides knowledge and emphasizes the maps’ precision, ensuring accurate and efficient data use in the future. Using a confusion matrix, we computed several metrics to assess the accuracy of classification. These metrics include omission error, commission error, overall accuracy, user’s accuracy, producer’s accuracy, and the kappa coefficient. GEE uses error matrix and producer/user accuracy methods to assess classifier accuracy. The producer’s accuracy determines the probability of correctly identifying a specific ground characteristic. We compute this metric by dividing the number of correctly classified pixels in each category by the total number of pixels sampled in that category.

To evaluate each classifier’s performance, we use overall accuracy (OA) and assess the impact of sampling techniques on classifier performance. Overall accuracy is the most commonly used metric because it allows for accurate interpretation and estimation. It shows the proportion of test data that the classifier correctly classified. Furthermore, we assess the class-level performance of a specific classifier using the confusion matrix, user accuracy, and producer accuracy. The user’s accuracy quantifies the likelihood that a pixel designated as a particular class on the map genuinely corresponds to that class. We determine it by dividing the number of accurately identified pixels in each category by the total number of pixels classified within that category. Cohen’s kappa coefficient is a technique for assessing accuracy in a discrete multivariate framework. This coefficient, despite the non-random assignment of pixels in image classification, takes into account the impact of random chance in assessing classification accuracy. It quantifies the classification’s improvement relative to the random allocation of pixels to their corresponding groups. Accurate assessment techniques such as overall accuracy, user accuracy, producer accuracy, Kappa statistics, and dependable tools such as Google Earth Engine, Landsat imagery, XGBoost, Landscape Metrix, and QGIS v 3.42.0 are critical. These methods and approaches allow for accurate and comprehensive assessments of LULC change, as well as urban expansion monitoring for sustainable development and environmental management.

#### Landscape metrics analysis

In urban environments, land use and land cover patches can have a variety of spatial configurations, such as size, shape, and connectivity. After classifying, identifying, and analyzing urban land use types, we performed the landscape metrics analysis on the supervised classification images from 1994 and 2022, which corresponded to the start (1994) and the end (2022) of the study. We calculated the following metrics using the Landscape Metrics Plug-in on QGIS v 3.42.0: landscape proportion, patch density, largest patch area, mean patch area, and number of patches. We used the LULC maps obtained from satellite data interpretation with landscape analysis tools to examine the structural properties of the various cover types. We calculated the landscape metrics for each LULC class.

These metrics uniquely contribute to the study’s objectives in several ways. Landscape proportion helps in understanding the relative abundance of different land cover types within the urban environment, crucial for assessing the extent of urban growth and the balance between built-up areas and natural landscapes. Patch density indicates the number of discrete land cover patches per unit area, essential for evaluating the degree of urbanization and its impact on ecological connectivity. The largest patch area metric identifies the size of the largest contiguous land cover patch, useful for assessing the dominance of certain land cover types and their ecological significance. Mean patch area provides an average size of land cover patches, offering insights into the typical scale of urban and natural patches, which helps in understanding the spatial structure of the urban landscape and its implications for ecological health. The total number of patches reflects landscape fragmentation, indicating more fragmented and heterogeneous landscapes that can affect urban growth patterns and ecological processes. By employing these metrics, we can effectively assess changes in land use and land cover, providing valuable insights into urban growth patterns and their ecological impacts, directly addressing our specific research objectives. To help readers understand the practical implications of these metrics, we visualized them using various graphical representations and maps. Thematic maps displayed the spatial distribution of different land cover types and their changes over time. These visualizations enhance the interpretability of the metrics and allow for a more intuitive understanding of urban growth patterns and their ecological implications.

#### Change detection analysis and transition potential modelling

Change detection analysis is instrumental in identifying and delineating geographical alterations across diverse land use categories. It entails evaluating the changes that occur in a region, object, or phenomenon over time. We performed a post-classification analysis utilizing classified images from 1994 to 2022, making it easier to examine alterations and calculate the area occupied by different land covers. Change detection analysis facilitates the identification and recognition of geographical alterations across diverse land use classifications. To make a LULC change map, we had to figure out how things changed over time and space and how LULC changed between the study periods (1994–2022) using the QGIS v 3.42.0 CA model Modules of Land Use Change Evaluation (MOLUSCE). We created an area change and transition probability matrix with rows and columns of landscape categories in the start and end years. We used an ANN multilayer perceptron to represent transition potentials (Muhammad et al., 2022).

We used a new QGIS v 3.42.0 plugin named MOLUSCE, which includes a transition probability matrix, to estimate potential changes in land use and land cover (LULC). The plugin is built using the Cellular Automata (CA) model. MOLUSCE is an independent tool for land use and land cover analysis, offering unique features not found in other prediction models. It supports multi-objective optimization, allowing it to balance competing objectives such as increasing agricultural productivity while minimizing environmental impacts and protecting green spaces. Gidey et al.^3^ identified several prevalent models for predicting future land cover, including cellular automata, Markov chains, artificial neural networks, and binary logistic regression. Yeasmin et al. (2025) reported that the artificial neural network model was used due to its broader and more successful applicability in recent years compared to other models. We generated a transition matrix, which illustrates the changes in class percentages compared to other classes^19^. We further calculated the area of the classes (water body, built-up, vegetated areas, bare land, and mining areas) and produced a transition matrix. Based on the transitional matrix, we estimated future land use and land cover for 2030. This produced a map showing the five classes and how they will transform by the year 2030. The MOLUSCE plugin provides a kappa validation technique and compares actual and projected LULC images.

The Artificial Neural Network (ANN) model was chosen over other models like Markov chains and logistic regression for several reasons. ANN models have demonstrated higher predictive accuracy in LULC change studies compared to Markov chains and logistic regression due to their ability to capture complex, non-linear relationships between variables^20^. Additionally, ANNs are highly flexible and can adapt to various types of data and patterns, making them suitable for modeling the diverse and dynamic nature of land cover changes^21^. ANNs can handle non-linear interactions and support multi-objective optimization, making them ideal for accurately predicting complex LULC changes while balancing competing objectives like agricultural productivity and environmental protection^22^. According to Mehra et al.^20^, ANN models have broader and more successful applicability in predicting LULC changes compared to other models, making them a preferred choice for long-term and large-scale environmental studies.

The MOLUSCE plugin in QGIS v 3.42.0 further enhances LULC prediction by incorporating a transition probability matrix and utilizing the Cellular Automata (CA) model. This combination allows for detailed spatiotemporal analysis and simulation of land cover changes, providing a more accurate and comprehensive understanding of LULC dynamics. MOLUSCE’s ability to integrate various spatial variables and its support for multi-objective optimization make it a powerful tool for addressing the complexities of LULC prediction^21^. By leveraging these advantages, the ANN model and MOLUSCE plugin provide a robust and reliable framework for predicting future land use and land cover changes.

## Results

### Analysis of urban expansion from 1994 to 2022

**Fig. 2:**
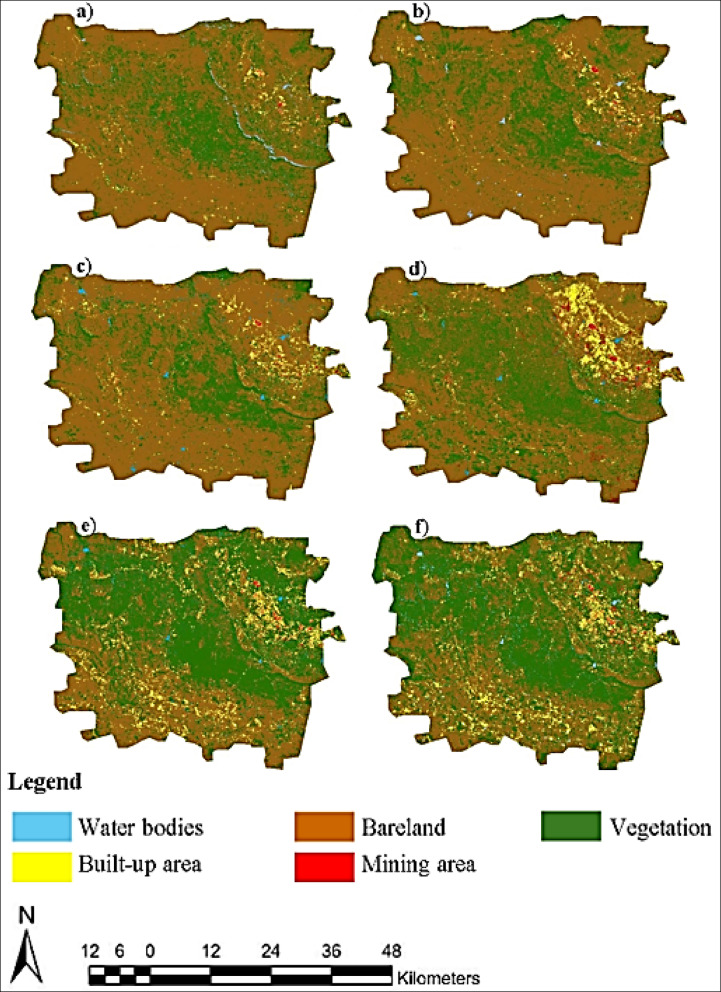
Historical trends of urban and non-urban land cover types in the study area based on an Extreme Gradient Boosting (XGBoost) model for 1994 (**a**), 1999 (**b**), 2000 (**c**), 2006 (**d**), 2012 (**e**), and 2022 (**f**), processed using QGIS v3.42.0 (open-source software, available for download at https://qgis.org/download/).

This study highlights a six-fold increase in built-up areas and a significant reduction in bare land, reflecting urbanization and ecological transformation over 28 years. The five major land cover types, such as water bodies, built-up areas, agricultural areas, bare land, and mining areas, were modeled with particular emphasis on the expansion of built-up areas over the last twenty-eight years (Fig. 2). When examining the various land cover types from 1994 to 2022 in the Rustenburg region, it becomes evident that there have been substantial changes and transformations.

In 1994, both water and built-up areas occupied a mere 1% of the landscape, indicating limited presence and development. However, by 2022, built-up areas had expanded to 6%, signifying substantial urban and suburban growth driven by population increase and infrastructure expansion. Meanwhile, the proportion of water areas remained constant at 1%, suggesting stability in aquatic features. The most striking change occurred in vegetated areas. In 1994, they accounted for 25% of the landscape, reflecting extensive natural cover like forests, meadows, and agricultural lands. By 2022, this category had expanded significantly, covering 51% of the region. This demonstrates the area’s ecological richness, intensified agricultural activity, and potential reforestation efforts. In contrast, bare land, which dominated in 1994 at 73%, decreased to 41% by 2022. This shift implies changes in land use, potential reclamation efforts, or agricultural expansion. A notable development was the absence of mining areas in Rustenburg in 1994, which had transformed by 2022 to occupy 1% of the landscape. This suggests the emergence of mining activities, fueled by the region’s demand for mineral resources.

When we look at the aerial coverage of each land type in hectares between 1994 and 2022, the Rustenburg region underwent significant transformations. In 1994, water and built-up areas covered 88,303 and 125,763 hectares, respectively, representing a small portion of the landscape. In contrast, vegetated areas spanned 2,513,336 hectares, indicating thriving natural ecosystems. Bare land dominated with 7,327,452 hectares, while mining areas were the smallest at 16,323 hectares. By 2022, the landscape had changed dramatically. Vegetated areas remained dominant at 5,104,145 hectares, emphasizing the region’s ecological richness. Built-up areas had expanded to 586,017 hectares, driven by population growth and urbanization. Land use changes or reclamation efforts reduced bare land to 4,147,455 hectares. Water bodies covered 120,336 hectares, and mining areas grew to 113,224 hectares, indicating increased mining activity.

Fragmentation often leads to the loss of continuous habitats, negatively impacting wildlife, especially species that require large, undisturbed areas, resulting in a decline in biodiversity. Fragmented patches create more edges, altering the microclimate and increasing exposure to invasive species, pests, and diseases, further degrading habitat quality. Vegetated areas provide essential ecosystem services such as carbon sequestration, water filtration, and soil stabilization, but fragmentation disrupts these services, leading to increased erosion, reduced water quality, and higher carbon emissions. The absolute sizes of urban features like patch density, largest patch area, mean patch area, and number of patches show that the landscape of the studied region changed significantly between 1994 and 2022 in terms of land cover patches (Table 3). In 1994, the terrain was characterized by a fragmented urban environment with 26,111 patches of built-up areas, while 57,257 patches highlighted a diverse distribution of natural ecosystems in vegetated areas. Bare land exhibited 30,490 patches, emphasizing extensive fragmentation in undeveloped or scarcely vegetated regions. Water bodies, with 5,423 patches, represented the distribution of aquatic features. Mining areas, at 2,267 patches, showed less dispersed mining activities.

**Table 3:** Landscape matrix analysis of the study area from 1994 to 2022.

Land cover type	Land cover (ha)	Landscape proportion %	Patch density	Greatest patch area	Mean patch area	Number of patches
1994						
Water bodies	88,303	1	0.0005	5771	16.283	5423
Built-up	125,763	1	0.0026	4736	4.816	26,111
Vegetation	2,513,336	25	0.0057	1,120,683	43.896	57,257
Bare land	7,327,452	73	0.0030	6,775,966	240.323	39,490
Mining area	16,323	0	0.0002	2888	7.200	2267
2022						
Water bodies	120,336	1	0.0028	3751	4.322	27,845
Built-up area	586,017	6	0.0060	13,413	9.656	60,690
Vegetation	5,104,145	51	0.0101	3,708,001	49.982	102,119
Bare land	4,147,455	41	0.0803	2,042,082	51.253	80,921
Mining area	113,224	1	0.0023	3494	4.860	23,296
Total	10,071,177	100	–	–	–	–

In contrast, 2022 saw significant changes: water patches increased to 27,845, showing greater fragmentation of aquatic features; built-up areas increased to 60,690 patches, indicating large-scale urban growth; vegetated areas grew to 102,119 patches, reflecting ecological diversity and varied land management; bare land showed 80,921 patches, indicating large-scale fragmentation; and mining areas had 23,296 patches, showing widespread mining. These shifts emphasize the evolving landscape dynamics and the necessity for adaptive land management and sustainable practices to effectively address changing environmental and developmental challenges. The analysis of patch density for 1994 and 2022 unveils significant changes in land cover distribution and fragmentation in the studied region. In 1994, water had a low patch density (0.000538467), indicating scattered water bodies. Built-up areas showed moderate fragmentation (0.002592646), while vegetated areas (0.005685234) displayed a coherent distribution.

Bare land exhibited moderate fragmentation (0.003027452), and mining areas had a lower patch density (0.000225098). Contrastingly, in 2022, water’s patch density increased significantly (0.002764821), signaling greater fragmentation of aquatic features. Built-up areas expanded substantially (0.006026108), reflecting urban growth. Vegetated areas became more fragmented (0.010139728), as did bare land (0.00803491), potentially influenced by several factors. Mining areas showed a dispersed distribution (0.002313136). While patch density, mean patch area, and other metrics are discussed, their implications are not fully explained. For instance, increased fragmentation in vegetated areas can indicate several ecological challenges or management issues. According to Wilson et al.^23^ and Bogaert et al.^24^, increased fragmentation in vegetated areas can indicate several ecological challenges or management issues, such as hindering species movement, reducing genetic diversity, and complicating land management efforts, including the need to monitor and maintain fragmented patches, leading to isolated wildlife populations, increased vulnerability to invasive species, and higher resource demands for effective land management.

### Accuracy assessment

Table 4 shows the accuracy of the XGBoost model, and the results indicate that water bodies exhibit the highest accuracies (0.99 Producer’s, 0.98 User), signifying robust concordance between map classifications and ground truth. Vegetation also shows high accuracy (0.96 Producer’s, 0.95 User), indicating dependable classification. Producers may inaccurately classify certain bare land; however, users can consistently identify it, with a slightly lower producer’s accuracy (0.93) and a higher user accuracy (0.97). Built-up areas exhibit reduced accuracies (0.9 Producer’s, 0.92 User), likely due to intricate urban patterns. Mining regions show comparable accuracies (0.95 Producer’s, 0.92 User), highlighting difficulties in delineating dynamic mining operations. The overall accuracy of 0.95 indicates that 95% of the land cover classifications are correct, which is a significant achievement. This high degree of accuracy makes the classification model highly reliable. The Kappa statistic, valued at 0.93, quantifies the extent of agreement between the classified data and the ground truth, incorporating the possibility of random chance. A Kappa value of 0.93 signifies near-perfect agreement, demonstrating the exceptional performance of the classification model beyond random chance. These metrics collectively demonstrate that the XGB model is both accurate and reliable, providing high confidence in the outcomes for urban land use analysis and planning.

**Table 4:** Accuracy assessment of the study.

Land use type	Water bodies	Built-up	Vegetation	Bare land	Mining area	Total	User accuracy
Water bodies	**392**	0	8	0	0	400	0.98
Built-up	0	**368**	0	15	17	400	0.92
Vegetation	1	5	**379**	14	1	400	0.95
Bare land	0	8	3	**388**	1	400	0.97
Mining area	2	29	3	0	**366**	400	0.92
Total	395	410	393	417	385	2000	–
Producer’s accuracy	0.99	0.90	0.96	0.93	0.95	–	–
Overall accuracy	0.95						
Kappa statistics	0.93						

**Fig. 3:**
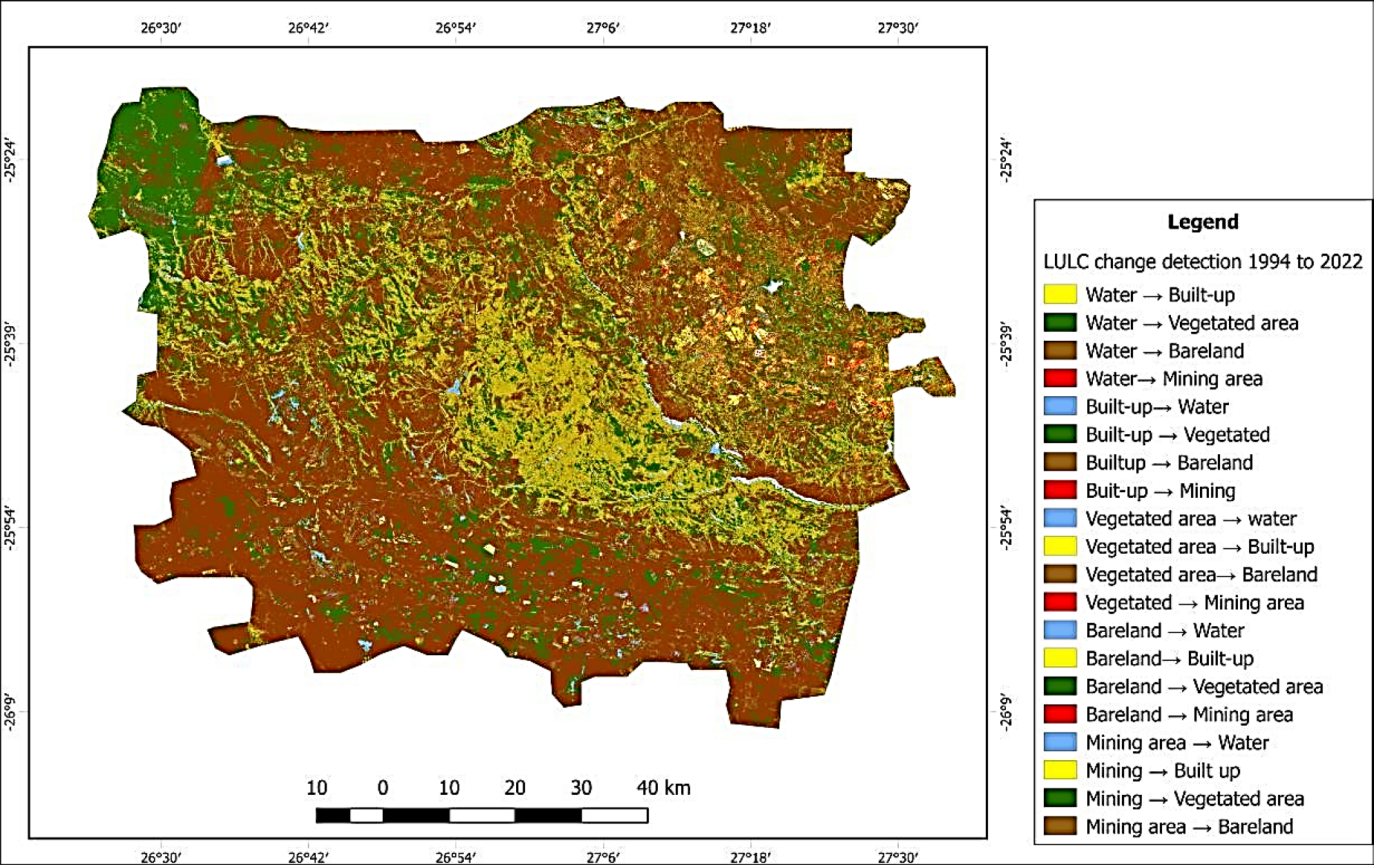
LULC Change detection map between 1994 and 2022, processed using QGIS v3.42.0 (open-source software, available for download at https://qgis.org/download/).

Figure 3 illustrates the changes that occurred in each land cover class between 1994 and 2022. The findings indicate a consistent increase in the built-up area from 1994 to 2022. In 1994, the water body area was 7,200 hectares. From 1994 to 2000, there was a significant reduction, with the area shrinking to 3,500 hectares. However, from 2000 to 2006, the water body area increased again, reaching 6,900 hectares. A further decrease occurred in 2012, with the area dropping to 4,000 hectares. Nonetheless, a substantial increase was observed from 2012 to 2018, with the water body expanding to 11,500 hectares. By 2022, the area had slightly decreased to 9,700 hectares, indicating a resurgence in the size of the water body.

Similarly, in 1994, the built-up area was 10,100 hectares. It steadily grew over the years, reaching 11,500 hectares in 2000 and 19,700 hectares in 2006. A substantial surge occurred in 2012, with the built-up area expanding to 36,300 hectares. This growth continued, reaching 44,500 hectares in 2018 and eventually 47,400 hectares in 2022, indicating significant urban expansion and infrastructure development throughout this period. From 1994 to 2006, the vegetated area remained steady, covering 202,800 hectares in 1994 and 173,200 hectares in 2006. However, starting in 2006, there was a considerable growth trend. The vegetated area expanded to 263,900 hectares in 2012, 396,000 hectares in 2018, and 411,900 hectares in 2022, indicating a significant increase in green and vegetated spaces over the years. The bare land area has changed over time. It was 591,100 hectares in 1994 and slightly increased to 595,300 hectares in 2000. The area continued to rise, reaching 609,500 hectares by 2006. However, there was a significant decrease from 2006 to 2018, with the area reducing to 354,700 hectares. By 2022, the bare land area further decreased to 334,400 hectares, reflecting changes in land use and probable urbanization.

While this analysis provides a detailed description of land use changes, it primarily focuses on data description and lacks an in-depth exploration of interactions between different land use types. For instance, the causal relationship between built-up area expansion and changes in vegetation or bare land remains underexplored. To better understand the spatial patterns of urban expansion, it is recommended to further analyze these dynamics. Investigating the drivers behind these transitions, such as economic development, policy changes, or environmental factors, could provide valuable insights into the underlying processes shaping land use changes.

### Prediction of the future urban expansion from 2022 to 2030

#### Analysis of land use change transition matrix from1994 to 2022

Table 5 presents the transition matrix, illustrating the changes in land cover classes between 1994 and 2022. Each row represents the land cover classes of 1994, while each column represents the land cover classes of 2022. The matrix values indicate the proportion or probability of a pixel transitioning from the class indicated by the row to the class indicated by the column. Analyzing the land use changes from 1994 to 2022 reveals various transformations in water areas. The majority, approximately 51.39%, of water in 1994 remained water in 2022, indicating stability in water bodies. However, a small proportion, about 0.93%, transitioned to built-up areas, and approximately 44.45% transformed into vegetated areas, potentially due to natural processes or shifts in land use. Additionally, around 2.70% of water areas shifted to bare land, likely linked to land development, while approximately 0.54% became mining areas due to mining activities.

**Table 5:** Transition matrix rates1994-2022.

Land cover type	Water bodies	Built-up	Vegetated area	Bare land	Mining area
Water bodies	0.513867	0.009332	0.444470	0.026964	0.005368
Built-up	0.03852	0.211302	0.183353	0.489277	0.085216
Vegetated Area	0.015418	0.017035	0.683610	0.277382	0.006555
Bare land	0.009486	0.026266	0.282737	0.674381	0.007130
Mining area	0.091527	0.357716	0.180849	0.102616	0.267291

Conversely, for built-up areas in 1994, about 3.85% converted to water, indicating urban expansion, while 21.13% remained built-up, showcasing some urban development stability. Roughly 18.34% transitioned to vegetated areas, potentially indicating urban greening or land use changes, and a substantial 48.93% transformed into bare land, reflecting land development. Around 8.52% shifted to mining areas due to mining-related activities.

While the analysis provides a detailed description of land use changes, it primarily focuses on data description and lacks an in-depth exploration of interactions between different land use types. For instance, the causal relationship between built-up area expansion and changes in vegetation or bare land remains underexplored. To better understand the spatial patterns of urban expansion, it is recommended to further analyze these dynamics. Investigating the drivers behind these transitions, such as economic development, policy changes, or environmental factors, could provide valuable insights into the underlying processes shaping land use changes.

**Fig. 4:**
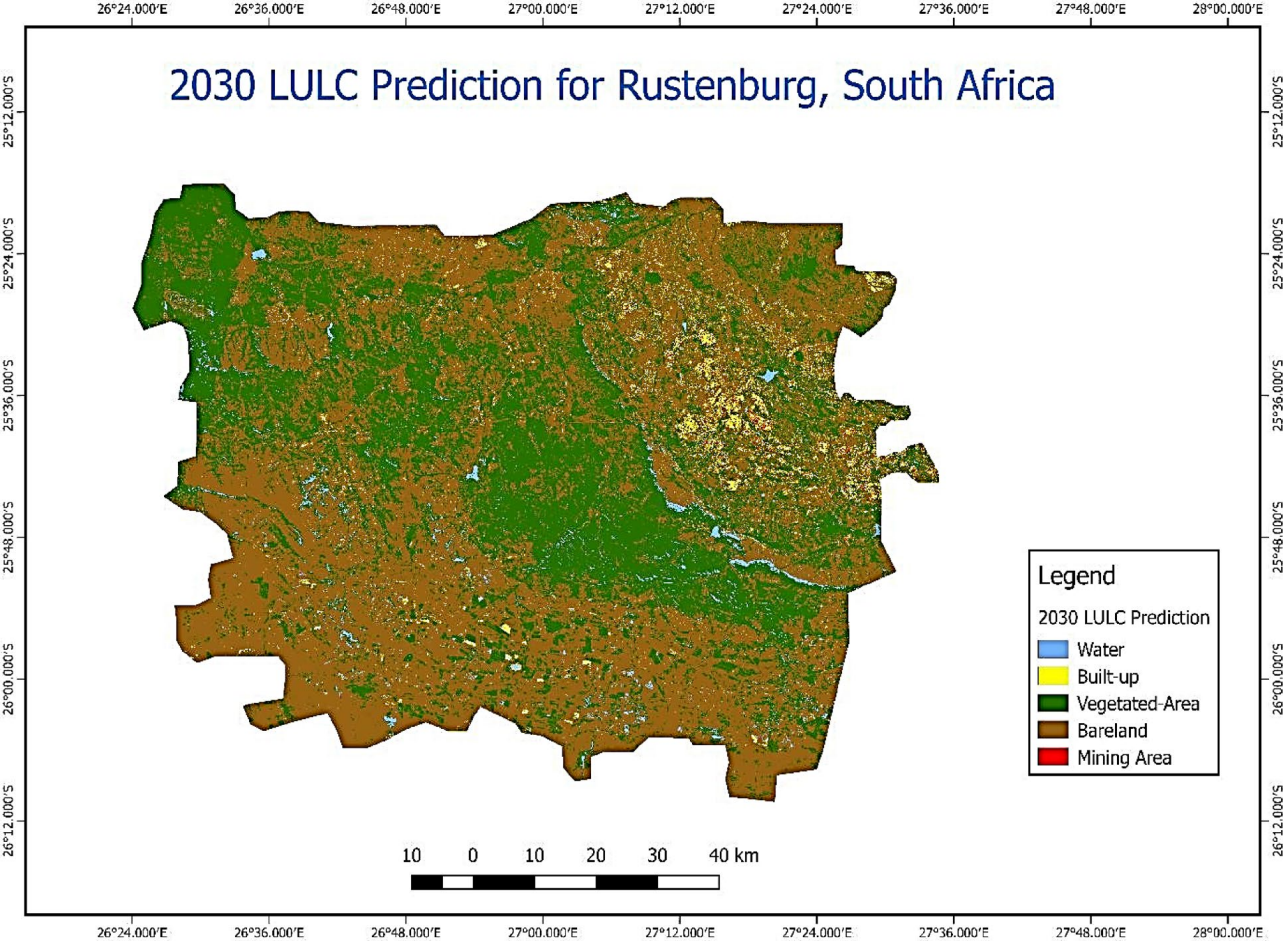
Future urban expansion in the City of Rustenburg, South Africa, processed using QGIS v3.42.0 (open-source software, available for download at https://qgis.org/download/).

We used the actual LULC layer of 2022 (Fig. 2f) to validate and predict future urban expansion in the study area, identifying five major LULC types (Fig. 4). The validation procedure employed the Kappa coefficient to estimate the agreement value. The 2030 LULC prediction’s validation accuracy resulted in an overall Kappa of 98%.

## Discussion

### Historical and future urban expansion and its associative effects in the city of Rustenburg from 1994 to 2030

Researchers have recognized the potential, strength, and effectiveness of remote sensing and geographic information systems in land use and land cover (LULC) studies (Mishra & Rai, 2016). Recent advancements in remote sensing and image classification allow for detecting changes in the land surface over larger areas at lower costs and with shorter processing times compared to conventional methods. According to Abdullah et al.^15^, recent research has utilized sophisticated classification algorithms such as random forests (RF), artificial neural networks (ANN), support vector machines (SVM), and extreme gradient boosting (XGBoost). These algorithms have been employed in various multi-spectral and hyperspectral image classification tasks, including LULC mapping and change detection studies. By using datasets featuring multiple time periods, change detection frameworks can quantify and qualitatively analyze the temporal effects of events. Attri et al.^25^ noted that these frameworks provide both qualitative and quantitative data on changes in characteristics, as well as information on their spatial distribution.

In this study, for mapping changes in LULC between 1994 and 2022, we used Landsat data (5TM, 7TM, and 8OLI) in conjunction with sophisticated classification algorithms. Specifically, we utilized XGBoost for an extensive evaluation of urban expansion in Rustenburg from 1994 to 2022. The landscape metrics assessment indicated an increase in built-up and mining areas. For instance, in 1994, the developed area encompassed 125,763 hectares. By 2022, the built-up area had significantly expanded to 586,017 hectares, increasing from 1 to 6%. These results demonstrate a substantial expansion of built-up areas over the last thirty years. Bett et al. (2013) corroborated these findings, focusing on urban sprawl observation in Rustenburg, South Africa, using a minimum distance supervised classification algorithm. Their data indicated a notable increase in the percentage of developed land from 1992 to 2009, from 2 to 9%, further underscoring the substantial growth of built-up areas over the past thirty years.

Previous studies, such as those by Attri et al.^25^ and Bett et al.^26^, have utilized various classification algorithms for LULC mapping and change detection. However, this study advances the field by employing XGBoost, a more sophisticated algorithm, for an extensive evaluation of urban expansion in Rustenburg from 1994 to 2022. Unlike earlier studies that used minimum distance supervised classification algorithms, this research leverages XGBoost, which offers higher accuracy and efficiency in processing large datasets. Additionally, the use of Landsat data (5TM, 7TM, and 8OLI) across multiple time periods provides a comprehensive view of changes in LULC over nearly three decades. The landscape metrics assessment indicates a significant increase in built-up and mining areas, with the developed area expanding from 125,763 hectares in 1994 to 586,017 hectares in 2022. This substantial growth underscores the effectiveness of the chosen methodology and data sources, contributing new insights and improvements to the field of LULC mapping and urban expansion analysis.

Furthermore, regional development strategies frequently encompass zoning regulations and economic incentives designed to stimulate urban growth. The construction of transportation infrastructure, such as new highways and public transit systems, can enhance the appeal of previously inaccessible areas for development. Additionally, population migration policies that encourage urbanization or address housing shortages can have a significant impact on urban expansion. Analyzing these factors at various spatial scales can offer a more thorough understanding of their effects on urban growth patterns. These socioeconomic factors likely played a crucial role in the observed expansion of built-up areas in Rustenburg, as evidenced by the significant increase in developed land from 1994 to 2022. The findings of this study have direct implications for urban planning strategies and mining regulations in Rustenburg. The substantial increase in built-up and mining areas highlights the need for sustainable urban planning practices to manage growth effectively. Urban planners should consider integrating green infrastructure, promoting conservation efforts, and regulating mining activities to minimize environmental degradation. Additionally, policymakers should focus on developing zoning regulations and economic incentives that balance development with environmental sustainability. By leveraging these insights, the Municipality of Rustenburg can enhance urban planning and monitoring processes, ensuring that future growth aligns with sustainability goals.

### Model validation

The increase in urbanization in Rustenburg is due to various factors, including natural population growth, post-apartheid urban development, an influx of migrants from adjacent provinces and countries, and the expanding mining sector^9^. Rustenburg distinctly illustrates the impact of apartheid-era migration limitations, with urban expansion focused in and around established townships and informal settlements. The mining and industrial activities in the region have led to an influx of labor into Rustenburg, significantly contributing to community development and alterations in the natural environment. Mining operations have rendered the soil desolate in previously vegetated areas and expanded mine worker settlements, adversely affecting the surrounding landscape. The proliferation of informal settlements in Rustenburg contributes to the observed high growth rate. As a platinum mining hub, Rustenburg’s urban expansion is closely tied to the increasing mining operations, underscoring a correlation between urbanization and mining. Mining typically affects population density as individuals migrate to mining regions for employment and resources.

### Landscape metrics and transition matrix analysis

The landscape metrics results shown in Table 3 indicate that the mining area encompassed 16,323 hectares in 1994. By 2022, the mining area had expanded to 113,224 hectares. The increase in mining activity in open areas has contributed to urbanization due to the influx of people seeking employment. The intensification of mining, agriculture, and urban development has significantly degraded the terrain. Urbanization has led to increased fragmentation, causing developed regions to expand outward. The largest patch area in bare land has changed, indicating that the land was more fragmented in 1994 compared to 2022. Extensive fragmentation allows for urban expansion on the periphery. The demand for additional housing, urban infrastructure, and services results in an escalation of patch density (PD). Newly developed areas have appeared on the peripheries of existing barren land, vegetation, and vegetated zones. However, the PD of certain land use classes has decreased, indicating that some patches are merging to create a more homogeneous environment.

We highlight the transition matrix as an essential instrument for clarifying the intricate dynamics of LULC. This analytical tool is critical for assessing net variations, the rate of change, and the specific characteristics of transitions that occur over two separate intervals. It helps to understand LULC changes and uncovers significant insights into the temporal patterns of these transformations, improving comprehension of this dynamic domain. Forecasting land use and land cover alterations is essential for promoting sustainable land management practices and mitigating negative impacts. The identification and forecasting of changes in LULC have become crucial in various fields, including modeling rural and urban development scenarios and identifying biodiversity hotspots for prioritizing conservation efforts. The predictive model anticipates the pattern and magnitude of forthcoming changes while also statistically and geographically documenting prior changes^19^. According to Muhammad et al.^27^, the MOLUSCE plugin is excellent for evaluating land use changes and can predict the chances of transitions, run simulations of the future, and examine forest and land use changes in both time and space. This approach is effective for managing substantial, ambiguous, or difficult-to-implement input data.

Since 1994, the developed area has expanded, and the 2030 LULC forecast suggests further growth is probable. The study revealed that in 1994, approximately 3.85% of developed areas transformed into water, signifying urban expansion, while 21.13% persisted as built-up, indicating a degree of stability in urban development. Prior research of a similar nature has also validated its importance. Alterations in LULC influence Earth’s ecosystems, leading to their deterioration. These changes are primarily manifested by a decrease in vegetation cover and alterations in the geographical range of LULC classes. According to Attri et al.^25^, this decrease initiates a chain reaction of negative environmental consequences, including loss of biodiversity, climate changes, changes in radiative forcing, contamination of natural ecosystems, altered hydrological patterns, and various other negative effects.

The landscape metrics and transition matrix results align with previous studies and Rustenburg-specific factors in several ways. The findings of increased fragmentation and urban sprawl are consistent with previous studies by Attri et al.^25^ and Bett et al.^26^ which also observed significant urban expansion and changes in land use patterns in Rustenburg. The use of sophisticated algorithms like XGBoost further validates the accuracy and efficiency of these findings. The results reflect Rustenburg’s unique socio-economic dynamics, including the impact of mining activities, population growth, and migration patterns. The significant increase in mining areas and the expansion of informal settlements around mines are specific to Rustenburg’s economic and social context, highlighting the correlation between urbanization and mining activities. The study’s findings underscore the need for sustainable urban planning practices in Rustenburg. The observed changes in landscape metrics and transition matrix values provide valuable insights for policymakers to develop zoning regulations, economic incentives, and infrastructure projects that balance development with environmental sustainability. By integrating these specific values and metrics, the study offers a comprehensive understanding of urban expansion and its associative effects in Rustenburg, aligning with both previous research and the city’s unique factors.

The utilization of Rustenburg’s resources has markedly augmented the city’s population as individuals flock in search of job prospects. The expansion of informal communities around mines has led to deteriorating social and physical conditions, such as a lack of essential services, increased crime rates, and higher rates of illness. Rustenburg, South Africa, is experiencing urbanization, and the findings of this study indicate that development has implications for urban development and environmental management. To gain a deeper insight into the Earth’s surface, multispectral satellite data detects changes in vegetation, land use/land cover, and urbanization, using dry month images to avoid seasonal effects^28–30^. This provides a foundation for future research and contributes to the formulation of policy decisions regarding urban expansion management. Using these findings, policymakers and other stakeholders can make informed decisions to ensure sustainable and equitable urban development. We will achieve this through an analysis of the factors driving urbanization, the patterns of urbanization, and the potential impacts of these factors.

## Conclusion

The study conducted in Rustenburg, South Africa, highlights the dramatic urban expansion from 1994 to 2030. Specifically, the findings reveal significant increases in vegetated areas to 5,104,145 hectares, built-up areas to 586,017 hectares, and mining areas to 113,224 hectares by 2022. Consequently, this growth has led to extensive fragmentation of natural ecosystems and significant environmental impacts, including biodiversity loss, climate change, and ecosystem contamination. Moreover, the rapid expansion of mining areas underscores the need for sustainable land management practices to mitigate adverse effects on the environment. The urban expansion in Rustenburg has profound implications for environmental sustainability and urban planning. The significant increase in built-up and mining areas has resulted in the fragmentation of natural ecosystems, leading to biodiversity loss and ecosystem contamination. These changes highlight the urgent need for informed policy decisions to ensure sustainable and equitable development. To mitigate the adverse effects of urbanization, it is crucial to implement sustainable land management practices. Policymakers should focus on integrating green infrastructure, promoting conservation efforts, and regulating mining activities to minimize environmental degradation. Additionally, the Municipality of Rustenburg can leverage these findings to enhance urban planning and monitoring processes, ensuring that future growth aligns with sustainability goals. Future research should incorporate higher-resolution satellite imagery (e.g., Pléiades Neo, WorldView-3, WorldView-4, RapidEye, etc.) to provide more detailed analyses of land use changes. Expanding the geographic scope to include multiple cities will offer comparative insights and broader applicability of the findings. Integrating socioeconomic data will help understand the drivers of urbanization and its impacts on local communities. Developing dynamic models that consider policy, and economic changes will enable more accurate predictions of future urban growth. Longitudinal studies are essential to monitor long-term trends and assess the effectiveness of implemented policies. By incorporating these additional data sources, future studies can provide a more comprehensive understanding of urbanization’s impacts and support the development of targeted strategies for sustainable urban growth.

## Data Availability

Data is provided within the manuscript or supplementary information files.
